# Chloroplast and Nuclear Genetic Diversity Explain the Limited Distribution of Endangered and Endemic *Thuja sutchuenensis* in China

**DOI:** 10.3389/fgene.2021.801229

**Published:** 2021-12-23

**Authors:** Zhi Yao, Xinyu Wang, Kailai Wang, Wenhao Yu, Purong Deng, Jinyi Dong, Yonghua Li, Kaifeng Cui, Yongbo Liu

**Affiliations:** ^1^ State Environmental Protection Key Laboratory of Regional Eco-Process and Function Assessment, Chinese Research Academy of Environmental Sciences, Beijing, China; ^2^ Hunan Provincial Collaborative Innovation Center for Field Weeds Control, Hunan University of Humanities, Science and Technology, Loudi, China; ^3^ Changbai Mountain Academy of Sciences, Joint Key Laboratory of Community and Biodiversity for Jilin Province and Changbai Mountain, Jilin, China

**Keywords:** chloroplast SSR, genetic diversity, limited distribution, narrow-ranged species, nuclear RAD-seq, Thuja sutchuenensis Franch

## Abstract

Narrow-ranged species face challenges from natural disasters and human activities, and to address why species distributes only in a limited region is of great significance. Here we investigated the genetic diversity, gene flow, and genetic differentiation in six wild and three cultivated populations of *Thuja sutchuenensis*, a species that survive only in the Daba mountain chain, using chloroplast simple sequence repeats (cpSSR) and nuclear restriction site-associated DNA sequencing (nRAD-seq). Wild *T. sutchuenensis* populations were from a common ancestral population at 203 ka, indicating they reached the Daba mountain chain before the start of population contraction at the Last Interglacial (LIG, ∼120–140 ka). *T. sutchuenensis* populations showed relatively high chloroplast but low nuclear genetic diversity. The genetic differentiation of nRAD-seq in any pairwise comparisons were low, while the cpSSR genetic differentiation values varied with pairwise comparisons of populations. High gene flow and low genetic differentiation resulted in a weak isolation-by-distance effect. The genetic diversity and differentiation of *T. sutchuenensis* explained its survival in the Daba mountain chain, while its narrow ecological niche from the relatively isolated and unique environment in the “refugia” limited its distribution.

## 1 Introduction

In the context of biodiversity loss worldwide, it is critical to conserve endangered species that are generally narrow ranged (Chau et al., 2013). The plant species with extremely small populations (PSESP) is proposed as a conservation concept to protect them (Yang et al., 2020). These species face extinction risk at any time because their population size is below a stable survival limit and suffers long-term interference and stress from external factors (Yang et al., 2020). The basis for species to adapt to environmental changes depends on the genetic diversity and differentiation, and even distribution area of populations (Jordan et al., 2019). Besides determining the ability to adapt to the environment, genetic diversity is the basis for maintaining long-term stability of the entire ecosystem (Hamrick et al., 1992). Species with small population size or small geographic ranges generally tend to result in low genetic diversity due to genetic drift, reduced gene flow, and increased genetic divergence (Ellstrand and Elam, 1993), which result in a low adaptability, reproductive ability, and disease resistance of species (Hamrick et al., 1992). Although there has been a debate, low genetic diversity generally increases extinction risk (Saccheri et al., 1998). Genetic variations or polymorphisms contribute to the viability and evolutionary potential of natural populations (Frankham and Briscoe, 2010); therefore, the assessment of genetic diversity is crucial in understanding the evolutionary history of endangered species and designing effective conservation and management methods (Cohen et al., 1991; Allendorf and Luikart, 2009; Ouborg, 2010).

The population demographic history is generally influenced by tectonic events and climatic change that, therefore, are key factors influencing patterns of genetic diversity (Sun et al., 2015). These tectonic and climatic events cause contraction–expansion of effective population size, thereby forming the current genetic structure (Bai et al., 2018). The extremity, variability, or stress of environmental factors can exacerbate not only population size but also latent genetic issues such as inbreeding, accumulated genetic load, and genetic variation (Fox and Reed, 2011). Thus, genetic diversity and structure of a species are indicators of life forms, reproductive systems, seed transmission mechanisms, evolutionary history, climatic factors, and human interference (Hamrick and Godt, 1996).

Here, we study the genetic diversity and structure of *Thuja sutchuenensis* Franch, an endangered species endemic to the Daba Mountains in northwestern Chongqing Municipality and eastern Sichuan Province, China (Xiang et al., 2002; Tang et al., 2015). Cooling and drying climate in the Quaternary forced *T. sutchuenensis* southward and restricted in the Daba mountains (Cui et al., 2015). When did *T. sutchuenensis* arrive at the Daba mountains, and can it survive only in this restricted region? Tang et al. (2015) found *T. sutchuenensis* populations distributed on limestone cliffs or steep slopes where there is lack of competition from other species. To address the endangered mechanisms of *T. sutchuenensis* and its limited distribution range in the Daba mountains as a “refugia,” wild and cultivated populations were genotyped using chloroplast simple sequence repeats (cpSSR) and nuclear restriction-site associated DNA sequence (nRAD-seq). Previous studies showed inconsistent results for *T. sutchuenensis* populations from different methods, high genetic diversity with ISSR (Liu et al., 2013), and low genetic diversity with six single-copy nuclear loci (Qin et al., 2020).

## 2 Material and Methods

### 2.1 Plant Materials Sampling

**FIGURE 1:**
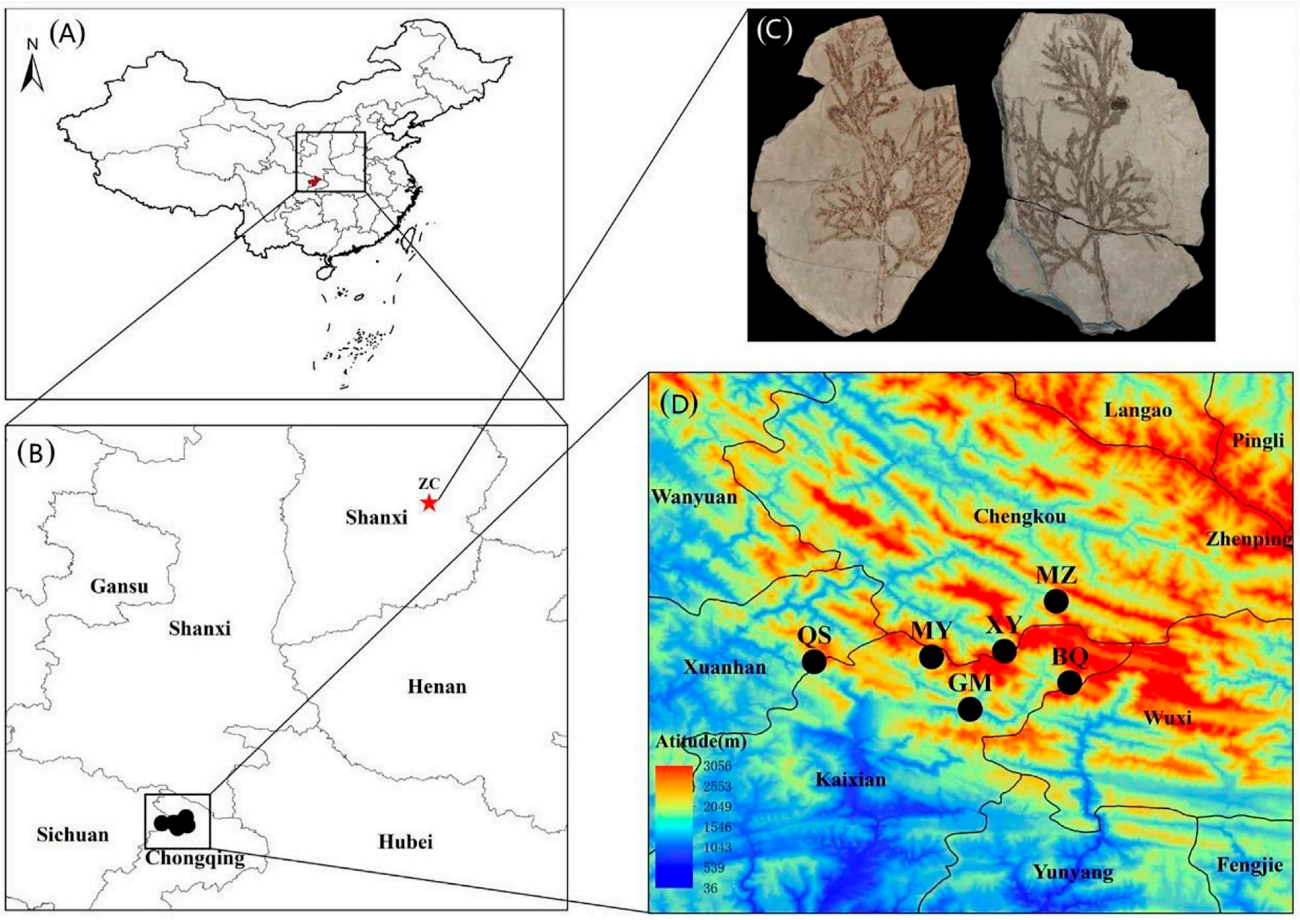
The distribution area and sampling sites of *Thuja sutchuenensis* in China. **(A)** and **(B)**, The locations of current *T. sutchuenensis* populations and *T. sutchuenensis* fossils in China. **(C)**, A picture of *T. sutchuenensis* fossils, which were found in Zhangcun, Shanxi province (36°58′N; 112°51′E), was quoted from Cui (Cui et al., 2015). **(D)**, Sampling populations of *T. sutchuenensis* in the Daba Mountains wild populations, with MZ and XY from the Chengkou County, and MY, GM, and BQ from the Kaixian County, Chongqing Municipality, as well as QS from the Xuanhan County, Sichuan Province.

*Thuja sutchuenensis*, a monoecious and evergreen coniferous tree, distributed in the Chongqing Municipality and Sichuan Province, Southwest China (Xiang et al., 2002; Cui et al., 2015) (Figure 1). We sampled 375 plants from six wild and three cultivated *T. sutchuenensis* populations, and recorded longitude, latitude, and altitude of the *T. sutchuenensis* plants (Table 1). We sampled individuals around 5 m from each other whenever possible. A total of ∼50 g fresh leaves per plant of *T. sutchuenensis* was sampled and dried in allochronic silica gel.

**TABLE 1:** Wild and cultivated *Thuja sutchuenensis* populations in the Daba Mountains.

Populations	Altitude (m)	Sampling individuals	Latitude	Longitude
Wild populations				
Baiquan (BQ)	1,597	30	31.5963	108.8369
Minzhong (MZ)	1,866	44	31.7321	108.8146
Guanmian (GM)	2,216	60	31.5522	108.6721
Xianyi (XY)	1,571	25	31.6488	108.7286
Qishu (QS)	1,583	20	31.6312	108.4131
Mingyue (MY)	2,003	20	31.6395	108.6079
Cultivated populations				
GMR		32	31.6024	108.6721
XYR		77	31.6494	108.7327
BQR		32	31.5965	108.8366

Note. Abbreviations: BQR, a cultivated population in the Baiquan County; GMR, a cultivated population in the Guanmian County; XYR, a cultivated population in the Xianyi County.

*Thuja koraiensis* (CYB) and *Platycladus orientalis* (ZYB), as two outgroups, were sampled from the Changbai Mountains and the Institute of Botany, CAS, respectively.

### 2.2 Chloroplast Simple Sequence Repeat Identification and Marker Design

Whole-chloroplast genome DNA of *T. sutchuenensis* was extracted from 25 mg of dry leaves using a Hi-DNA Secure Plant Kit (TIANGEN Biotech Co., Ltd.) based on a modified version of the SDS method (Yuan et al., 2012). The motifs containing the SSR loci were identified using MISA (http://pgrc.ipk-gatersleben.de/misa/). The motifs of trinucleotide, tetranucleotide, pentanucleotide, and hexanucleotide repeats included at least 5, 4, 4, and 4 repeats, respectively. SSR primers were designed using Primer 3.0 (https://sourceforge.net/projects/primer3/). The SSR primers were designed as follows: 18–24 bp in length, 40%–60% GC content, 55°C–65°C annealing temperature, and 100–300 bp PCR product. Furthermore, 400 SSR loci were selected and primers of 140 loci were screened in 20 individuals. Finally, 30 microsatellite repeats (SSR) were selected from the 140 loci to detect the polymorphic site of *T. sutchuenensis* populations (Supplementary Table S1), and the primers were synthesized by Sangon Biotech Co., Ltd. (Shanghai, China). Forward primers were labeled with four kinds of 5′-fluorescein bases (FAM, HEX, TRAMA, or ROX). The multiplex PCR (Supplementary Table S2) was run on a BIO-RAD T100™ Thermal Cycler. Detection of multiplex PCR products was carried out with an ABI 3730xl DNA analyzer (Sangon Biotech Co. Ltd.), setting a default range of standard length ±40 bp. The detection bands of 30 markers were scored using GeneMarker version 1.91 (size standard: GS500).

### 2.3 Nuclear Restriction Site-Associated DNA Sequencing

A total of 100 ng genomic DNA per sample was digested with two restriction enzymes, EcoRI, and PstI (New England Biolabs, Beverly, USA) at 37°C for 8 h. The restriction enzymes were then inactivated by heating at 65°C for 20 min. After ligation individually with barcoded EcoRI adapter and universal PstI adapter with T4 DNA ligase for each sample at 16°C for 8 h, the reaction was stopped by heating at 65°C for 20 min. The ligation products of all samples were equally pooled and size selected into 300–500 bp fragments using the agarose gel electrophoresis. After manipulating gel purification, derived fragments were used as templates (about 30 ng) for PCR amplification *via* 25 cycles with EcoRI and PstI adapter universal primers using PrimeStar Max DNA Polymerase (Takara, Dalian, China). Finally, the amplicons were size selected once more into 350–500 bp fragments with the method mentioned above. The resulting ddRAD library was sent to Guangzhou Jierui Biotechnology Company, LTD. (Guangzhou, China) and sequenced on the Illumina Novaseq platform using 150 nt with paired-end mode.

We used *process_radtags* module in the Stacks-2.4 (Catchen et al., 2011) to demultiplex the raw data, with default parameters. The reads were trimmed to 135-bp length to remove low-quality nucleotides at the 3′ end of each read. Finally, each end of the retained reads was treated as an independent locus and combined together. The *ustacks* module was employed to cluster the reads into exactly matching stacks, setting m = 2 as the minimum depth of coverage (m) and M = 12 as the maximum distance allowed between stacks within an individual. We then used the *cstacks* module to build the catalogs for all samples with n = 12 as the maximum number of mismatches allowed between individuals. The *Sstacks* module generated alignment results for each individual against the catalog using default parameters. In the *populations* module, we set *p* = 8 and r = 0.6 to call consensus SNP among all 137 samples (15 individuals per population).

### 2.4 Statistical Analysis

We employed Micro-Checker 2.2 to detect null alleles, scoring errors and allele dropout (Oosterhout et al., 2010), and no scoring errors or allele dropout were detected. Linkage disequilibrium and Hardy–Weinberg equilibrium were tested in Arlequin v. 3.5 (Excoffier and Lischer, 2010). Three of the 30 loci were null alleles, and a pattern of linkage disequilibrium was found at seven loci (*p *> 0.05) (Supplementary Table S3). Genetic diversity indices of cpSSR, including the number of alleles (*Na*), effective number of alleles (*Ne*), observed heterozygosity (*Ho*), expected heterozygosity (*He*), and inbreeding coefficient (*F*_*IS*_), were calculated for all samples from nine populations by GenAlEx version 6.503 (Peakall and Smouse, 2006).

Genetic diversity indices of nRAD-seq, including the inbreeding coefficient (*F*_*IS*_), nucleotide diversity (*Pi*), observed heterozygosity (*Ho*), and expected heterozygosity (*He*), were calculated using the *populations* module in Stacks (Catchen et al., 2011). A variant call format (VCF) file was generated using the *populations* program in Stacks (Catchen et al., 2011). A maximum likelihood (ML) tree of six wild and three cultivated populations (90 individuals) was constructed with 1,000 bootstraps using the SNPhylo software (Tae-Ho et al., 2014).

We separately calculated genetic differentiation and gene flow using GenAlEx for cpSSR data (Peakall and Smouse, 2006). The genetic differentiation coefficient (*F*_*ST*_) was calculated by analysis of molecular variance (AMOVA) (Excoffier et al., 1992). The significance of *F*_*ST*_ values was tested with 999 permutations, followed by Bonferroni correction. Gene flow (*Nm*) was estimated based on the formula $Nm=\left(1-F_{ST}\right)/4F_{ST}$ . Hierarchical AMOVA with 999 permutations was constructed to determine the genetic differentiation between populations (Peakall and Smouse, 2006). *F*_ST_ values of nRAD-seq data were calculated with the *populations* module and tested based on 1,000 permutations with Arlequin v3.5 (Excoffier and Lischer, 2010). AMOVA was also conducted to assess genetic differentiations within wild or cultivated populations. The analyses were conducted in Arlequin 3.5.2.1 (Excoffier and Lischer, 2010), and the significance level for the variance components was computed using 1,000 permutations. Summary outputs were displayed to get population structure with the program Distruct 2.1 (Rosenberg, 2004). PCoA analysis was conducted using the Ad genet package in R (Jombart, 2008).

To test whether genetic differentiation related with geographic distance, we performed Mantel test with 999 permutations using *F*_*ST*_/(1-*F*_*ST*_) and logarithmic geographic distance (Smouse, 1986). The pairwise matrix of geographic distances was calculated based on geographic coordinates, which were identified by a GPS device (Garmin Oregon 450). All calculations above were performed by GenAlEx (Peakall and Smouse, 2006).

To infer population structure, Bayesian clustering analysis was conducted in Structure version 2.3.4 (Pritchard et al., 2000). We set the number of groups (K) from 1 to 9 with 10 independent runs using an admixture ancestry model and 1,000,000-step Markov chain Monte Carlo (MCMC) replicates after a 500,000-step burn-in for each run. The best K value was inferred by delta K in Structure Harvester (Earl and Vonholdt, 2012). A cluster analysis based on genetic distance was performed by the unweighted pair-group method with arithmetic mean (UPGMA) approach in MEGA 6.0 (Tamura et al., 2013). We used the TreeMix program to detect historical migration among wild populations (Pickrell and Pritchard, 2012).

BOTTLENECK was used to examine recent bottleneck events (Cornuet and Luikart, 1996), under the TPM (two phase model), which allows multiple-step mutations (Peterson et al., 1994), with a 10% infinite allele mutation, 90% stepwise mutation model, and 1,000 replicates (Wang et al., 2014). The significance of heterozygote excess was determined by Wilcoxon’s signed-rank test (*p *< 0.05). Departures from mutation–drift equilibrium were detected by a mode-shift test (Luikart et al., 1998).

**FIGURE 2:**
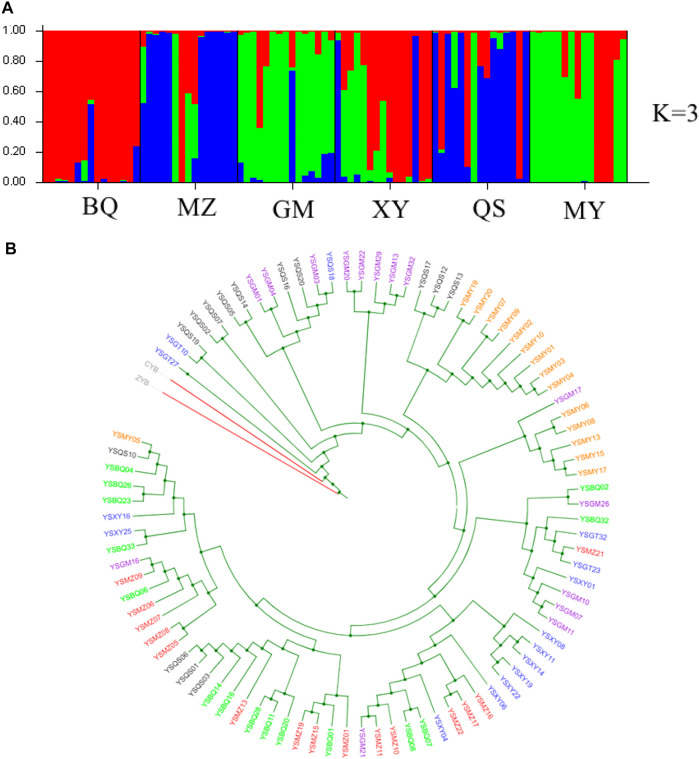
Genetic structure and phylogenetic tree of six wild *T. sutchuenensis* populations. **(A)** Population structure bar plots from nuclear restriction site-associated DNA sequencing (nRAD-seq) data show the clustering of samples into three clusters (best delta-K). Each vertical bar indicates an individual, and the height of each colored bar represents the proportion of assignment to that cluster. For population abbreviations, please see Figure 1. **(B)** Maximum-likelihood (ML) tree illustrates genetic relationships between six wild *T. sutchuenensis* populations based on SNPs from nRAD-seq. One color indicates one population. ZYB and CYB are outgroups.

To further investigate the demographic history of wild populations, we performed coalescent simulations of 15 evolutionary models (Supplementary Figure S1) in fastsimcoal 2 v2.5.2.2.21 (Excoffier and Foll, 2011). Based on the results of structure analysis, wild populations were clustered in three groups (best K = 3), Pop 1 (BQ and XY), Pop 2 (MZ and QS), and Pop 3 (MY and GM) (Figure 2; Supplementary Figure S2). The fastsimcoal software simulates these data following the Wright–Fisher model of evolution assuming neutrality of the genetic markers, without recombination within loci but free recombination between loci and random mating. We improved the performance of the models by reducing the number of estimated parameters (Excoffier et al., 2013), with the population parameters were calculated directly from the SNP data. Each run was performed with 200,000 simulations per likelihood estimation and 40 expectation–conditional maximization (ECM) cycles. The mutation rate was set as 1.0 × 10^−8^.

To test the local adaptations of *T. sutchuenensis*, we used two genotype–environment associations analysis to detect loci related with environmental factors. One approach is OutFLANK, inferring the distribution of *F*_*ST*_ to detect loci responsible for local adaptation based on the expected Chi-square (χ^2^) distribution of *F*_*ST*_ in the absence of selection (Whitlock and Lotterhos, 2015). We clipped the *F*_*ST*_ distribution to 5% and 10% using the minimum expected heterozygosity of 0.10 and an error discovery rate of less than 0.05. The second approach is using latent factor mixed model (LFMM) (Frichot et al., 2013), to identify the relationship between genetic polymorphism of single nucleotide and environmental factors, using the locus GIF (genomic inflation factor) associated with the environment. The “VCF2LFMM” algorithm in the package was used to convert the nucleotide diversity files in the VCF format into the 0,1 format files of LFMM (Frichot et al., 2013). Extracted values of bioclimatic factors corresponding to species distribution points were used as environmental variables, and LFMM was used to convert environmental variables into the “env” format. Using 100,000 scans, “Burn-In” was set to 50,000 as recommended. In order to effectively control false discovery rate (FDR), we selected the K value whose estimated value of λ is close to or slightly lower than 1.0. Since the test of K values in a wide range is usually ineffective, we chose K = 1–10. Sites associated with the environment were finally picked out. The climatic data were downloaded from the WorldClim database, including 19 bioclimatic variables (bio1 to bio19) with a spatial resolution of 30 arc-seconds (http://worldclim.org) (Supplementary Table S4).

## 3 Results

### 3.1 Genetic Diversity

After all quality filters, 62,375,634 reads with no rad tag and 1,000,938 low-quality reads were discarded. We retained a total of 3,509,088,912 reads from the initial 3,609,108,036 raw reads, with an average of 2.63 Mb reads per sample. After trimming and clustering, we obtained loci number ranging at 6,933–395,804 bp and mean locus coverage across all samples was 72.64X from 32.69 to 135.05X, with an average length of 135 bp per loci and with 44.92% GC content. After the “cstacks” module processing, we obtained 4,886,198 catalogs and 6,119 SNPs.

Out of the 5,990 polymorphic SNPs loci, 2 (14063_15 and 3630303_6) and 1 (3630303_6) SNPs were considered as responsible for local adaptation by the OutFLANK and LFMM approaches, respectively. Because there is no existing reference genome for Cupressaceae, it is impossible to annotate these selected loci.

*T. sutchuenensis* populations showed high chloroplast genetic diversity (cpSSR) and low nuclear genetic diversity (nRAD-seq). The *Ho* and *He* of cpSSR in *T. sutchuenensis* populations were 0.43–0.92 and 0.23–0.53, respectively (Table 2). The *Ho* and *He* of cpSSR in BQ and QS populations were higher than in other wild populations (Table 2), while the nRAD-seq genetic diversity of *T. sutchuenensis* among populations were similar (*Ho* = 0.061–0.070, *He* = 0.078–0.087) (Table 3). The nucleotide diversity (*pi*) of *T. sutchuenensis* populations ranged from 0.082 to 0.092 for variant position and from 0.0053 to 0.0062 for all positions (Table 3).

**TABLE 2:** The characteristics of chloroplast genetic diversity (cpSSR) in *Thuja sutchuenensis* populations.

Populations	BQ^b^	MZ	GM	XY	QS	MY	GMR	XYR	BQR
Altitude (m)	1,597	1,866	2,216	1,571	1,583	2,003			
Latitude	31.5963	31.7343	31.5522	31.6856	31.6312	31.6189	31.7343	31.5780	31.6947
Longitude	108.8369	108.8102	108.6721	108.6632	108.4131	108.5651	108.8102	108.3747	108.6450
Sample size^a^	20	20	20	20	20	20	20	20	20
*Na* (SD)	3.233 (1.406) b	2.100 (1.729) ab	3.100 (1.583) b	3.000 (1.682) ab	3.033 (0.377) ab	1.933 (1.363) a	2.933 (1.437) ab	2.367 (0.999) ab	2.833 (0.950) ab
*Ne* (SD)	2.167 (0.334) d	1.532 (0.851) ab	1.888 (0.661) abcd	1.690 (0.692) abc	2.134 (0.313) cd	1.469 (0.598) a	2.165 (0.473) d	1.679 (0.505) ab	1.936 (0.333) bcd
*Ho* (SD)	0.920 (0.178) e	0.348 (0.428) ab	0.617 (0.396) bcd	0.441 (0.395) ab	0.925 (0.140) e	0.342 (0.424) a	0.888 (0.225) de	0.548 (0.418) abc	0.792 (0.275) cde
*He* (SD)	0.526 (0.085) c	0.233 (0.251) a	0.400 (0.220) bc	0.326 (0.224) ab	0.523 (0.618) c	0.226 (0.248) a	0.515 (0.123) c	0.342 (0.219) ab	0.465 (0.115) bc
*F*_*IS*_ (SD)	−0.742 (0.266) ab	−0.191 (0.493) d	−0.388 (0.460) cd	−0.200 (0.478) d	−0.773 (0.246) a	−0.171 (0.520) d	−0.709 (0.303) abc	−0.412 (0.460) bcd	−0.650 (0.345) abc

a Note. The 20 individuals were randomly selected from all samples to avoid bias due to sample size in calculating genetic diversity.

b For abbreviations of population names, please see Figure 1.

Different letters in the same column indicate significant difference at *p *< 0.05.

*Na*, number of alleles; *Ne*, effective number of alleles; *Ho*, observed heterozygosity; *He*, expected heterozygosity; *F*_*IS*_, fixation index.

**TABLE 3:** The characteristics of nuclear genetic diversity (nRAD-seq) in *T. sutchuenensis* populations.

Taxon	Populations^a^	*A*_P_	Polymorphic loci (%)	*H*_*O*_		*He*		*pi*		*F*_*IS*_	
				Variant positions	All positions	Variant positions	All positions	Variant positions	All positions	Variant positions	All positions
Wild *T. sutchuenensis*	BQ	293	0.23503	0.06531	0.00044	0.08404	0.00057	0.08815	0.0006	0.07798	0.00053
	MZ	189	0.22655	0.06057	0.00041	0.08639	0.00058	0.09087	0.00061	0.09676	0.00065
	GM	242	0.19884	0.06726	0.00043	0.07781	0.0005	0.08212	0.00053	0.04966	0.00032
	XY	237	0.21768	0.06965	0.00047	0.08092	0.00054	0.08523	0.00057	0.05364	0.00036
	QS	380	0.24302	0.06129	0.00041	0.08709	0.00059	0.09153	0.00062	0.09443	0.00064
	MY	300	0.22486	0.06413	0.00044	0.07865	0.00054	0.08204	0.00056	0.05785	0.0004
Cultivated *T. sutchuenensis*	GMR	143	0.22943	0.06893	0.00047	0.08211	0.00056	0.08554	0.00059	0.05682	0.00039
	XYR	205	0.22903	0.06575	0.00045	0.08191	0.00056	0.08539	0.00059	0.0646	0.00044
	BQR	176	0.23547	0.06619	0.00045	0.08434	0.00058	0.08807	0.0006	0.07467	0.00051
Outgroups	*Platycladus orientalis* (ZYB)	419	0.04327	0.0645	0.00043	0.03225	0.00022	0.0645	0.00043		
	*T. koraiensis* (CYB)	951	0.04649	0.06777	0.00046	0.03388	0.00023	0.06777	0.00046		

Note. *A*_P_, private allele number; *H*_*O*_, observed heterozygosity; *He*, expected heterozygosity; *pi*, nucleotide diversity; *F*_*IS*_, inbreeding coefficient.

a For population abbreviations, please see Figure 1.

The *Ho* and *He* of cpSSR in cultivated populations BQR and GMR were comparable with wild populations BQ and QS (Table 2). The high absolute values of cpSSR *F*_*IS*_ suggested a significant homozygous excess of *T. sutchuenensis* populations (Table 2), while the *F*_*IS*_ values of nRAD-seq were low (Table 3). The tested loci were not deviated from the Hardy–Weinberg equilibrium (HWE) (Supplementary Table S5). The wild QS population and the cultivated GMR and BQR populations experienced recent bottleneck events because their loci departures from the mutation–drift equilibrium in the allele frequency distribution (Supplementary Table S6).

### 3.2 Genetic Differentiation and Population Structure

The cpSSR genetic differentiation varied with the pairwise comparisons of populations (*F*_*ST*_ = 0.01–0.19; *Nm* = 1.12–22.48) (Table 4). The genetic differentiation (*F*_*ST*_) in pairwise comparisons of BQ-QS and MZ-MY-XY was low, with high gene flow (*Nm* = 7.13–10.62) (Table 4). The cultivated GMR and BQR populations and wild BQ-QS populations showed low pairwise *F*_*ST*_ values (*F*_*ST*_ = 0.01–0.03) and high gene flow (*Nm* = 5.56–22.48) (Table 4). The cultivated XYR population showed high cpSSR *F*_*ST*_ and low *Nm* from other populations (Table 4). However, the genetic differentiation of nRAD-seq in any pairwise comparisons were low (*F*_*ST*_ = 0.033–0.055) with high gene flow (*Nm* = 4.26–8.32) (Table 4). This indicates that *T. sutchuenensis* populations were genetically similar (Ewens, 1970), which was also supported by the analysis of molecular variance (AMOVA) that showed 81% of genetic variation within populations and 19% among populations (Supplementary Table S7).

**TABLE 4:** Matrix of pairwise *F*_*ST*_ and *Nm* coefficients of *T. sutchuenensis* populations from chloroplast simple sequence repeats (cpSSR) and nRAD-seq (values in parentheses).

	BQ	MZ	GM	XY	QS	MY	GMR	XYR	BQR
BQ		0.178 (0.044)	0.121 (0.047)	0.129 (0.039)	0.023 (0.047)	0.176 (0.044)	0.030 (0.033)	0.145 (0.038)	0.043 (0.035)
MZ	1.154 (5.375)		0.132 (0.055)	0.030 (0.050)	0.180 (0.055)	0.034 (0.051)	0.190 (0.043)	0.146 (0.044)	0.182 (0.043)
GM	1.816 (5.070)	1.644 (4.298)		0.068 (0.049)	0.127 (0.051)	0.123 (0.047)	0.138 (0.045)	0.125 (0.048)	0.133 (0.046)
XY	1.688 (6.096)	8.083 (4.768)	3.426 (4.816)		0.135 (0.049)	0.027 (0.045)	0.146 (0.036)	0.110 (0.041)	0.136 (0.039)
QS	10.620 (5.094)	1.139 (4.261)	1.719 (4.659)	1.602 (4.863)		0.180 (0.047)	0.011 (0.045)	0.159 (0.046)	0.028 (0.046)
MY	1.170 (5.482)	7.103 (4.690)	1.783 (5.028)	9.009 (5.277)	1.139 (5.075)		0.191 (0.040)	0.138 (0.043)	0.175 (0.043)
GMR	8.083 (7.243)	1.066 (5.571)	1.562 (5.313)	1.462 (6.621)	22.477 (5.291)	1.059 (5.958)		0.166 (0.035)	0.016 (0.029)
XYR	1.474 (6.252)	1.462 (5.369)	1.750 (4.914)	2.023 (5.859)	1.322 (5.139)	1.562 (5.600)	1.256 (6.990)		0.157 (0.037)
BQR	5.564 (6.829)	1.124 (5.572)	1.630 (5.161)	1.588 (6.117)	8.679 (5.217)	1.179 (5.557)	15.375 (8.324)	1.342 (6.425)	

Note. Top right matrix refers to pairwise genetic differentiation coefficient. Lower left matrix refers to pairwise gene flow coefficient.

The correlation between genetic and geographic distance was marginally significant (R^2^ = 0.029, *p* = 0.08) (Supplementary Figure S3), indicating a weak effect of isolation by distance.

The structure analysis of cpSSR showed two clusters (best delta K = 2) (Supplementary Figure S4), with one cluster of BQ, QS, GMR, and BQR populations and others clustered together. When K = 4 (the second top delta K) (Supplementary Figure S4), six wild populations clustered three groups and the population XYR cultivated from seeds 15 years ago differed from other populations. The nRAD-seq structure for the nine populations indicated gene flow between populations or originated from a common ancestor (Supplementary Figure S5). The structure analysis of nRAD-seq for six wild populations showed three clusters (best delta K = 3) (Figure 2A; Supplementary Figure S2). The results of UPGMA (Supplementary Figure S6), phylogenetic tree (Figure 2B), principal coordinate analysis (PCoA) (Supplementary Figure S7A), and principal component analysis (PCA) (Supplementary Figure S7B) confirmed the structure results from cpSSR and nRAD-seq, respectively.

### 3.3 Demographic History

**FIGURE 3:**
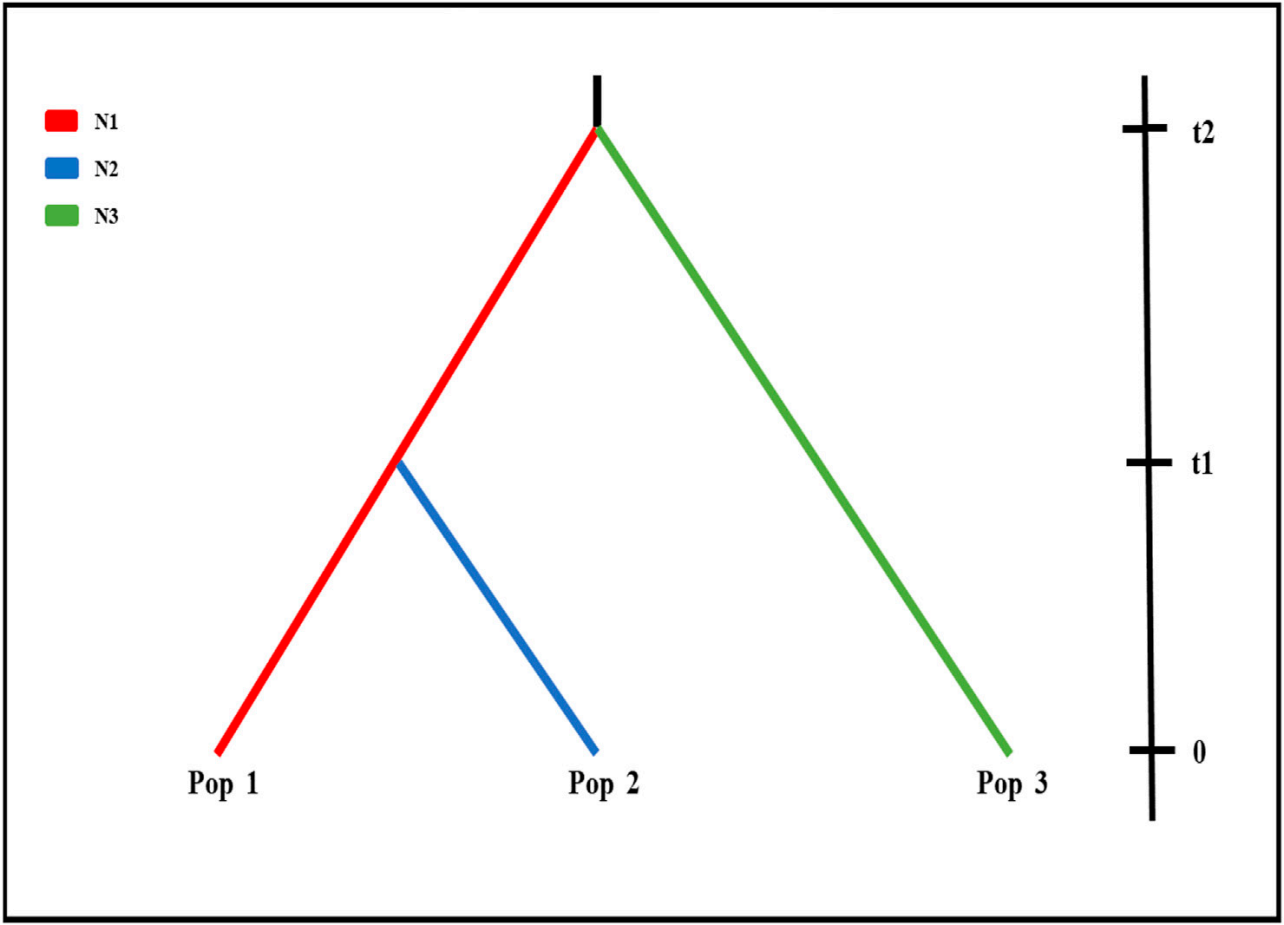
Demographic scenario tested in Fastsimcoal. Scenarios were demographic scenarios that were set to infer population history. Effective population size and divergence time for the three clusters (K = 3; Figure 2) for six wild populations using nRAD-seq data (see Supplementary Figure S1), i.e., BQ and XY populations (Pop 1, red line), MZ and QS populations (Pop 2, blue line), and GM and MY populations (Pop 3, green line). t2, divergence time between Pop 1 and Pop 3; t1, divergence time between Pop 1 and Pop 2.

Fastsimcoal results from nRAD-seq showed that the wild populations Pop 1 (BQ and XY) and Pop 3 (MY and GM) were from an ancestral population at ∼203 ka (Figure 2). An admixture event occurred at ∼12 ka between the Pop 1 and Pop 3 and formed the Pop 2 (MZ and QS) (Figure 2A, Figure 3; Supplementary Figure S2).

**FIGURE 4:**
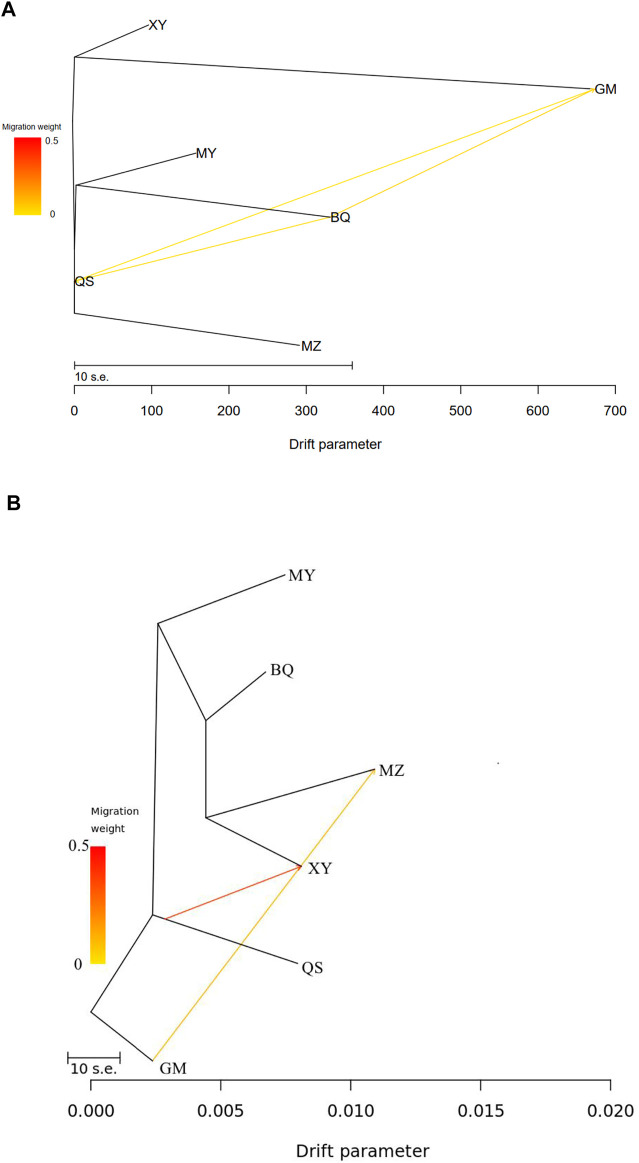
TreeMix results showing historical mitigation between the wild *T. sutchuenensis* populations. **(A)**, TreeMix results from cpSSR. **(B)**, TreeMix results from nRAD-seq. For population abbreviations, please see Figure 1.

TreeMix analysis of cpSSR revealed historic migrations between QS, BQ, and GM populations (Figure 4A; Supplementary Figures S8A, S9), while nRAD-seq data showed two migration events, from GM to MZ and from QS to XY (Figure 4B; Supplementary Figures S8B and S10).

## 4 Discussion

Assessing the genetic diversity and genetic structure of rare, endemic, and endangered species, i.e., narrow-ranged species, is not only the basis for developing scientific and rational conservation strategies (Joshi and Dhawan, 2007), but also an explanation of the limited distribution of the narrow-ranged species, such as wild plants with extremely small populations (PSESP) (Yang et al., 2017). We assessed the wild populations of *T. sutchuenensis* using chloroplast simple sequence repeats (cpSSR) and nuclear restriction site-associated DNA sequencing (nRAD-seq) and found relatively high chloroplast genetic diversity but low nuclear genetic diversity, which provides molecular explanation in its distribution in the Daba mountains as a “refugia”.

*T. sutchuenensis* was widely distributed in China in the late Pliocene based on its fossil in the Shanxi province (Figure 1C) (Cui et al., 2015), and its distributed area and population size decreased from the Last Interglacial (LIG, ∼120–140 ka) to the Last Glacial Maximum (LGM, ∼22 ka) (Qin et al., 2017). Our results found that *T. sutchuenensis* wild populations were from a common ancestral population at 203 ka, which means that *T. sutchuenensis* southward-migrated and reached the Daba mountains before the population concentration of *T. sutchuenensis*. MaxEnt results from Qin et al. (2017) found that the current distribution size is smaller than the past (LIG, LGM) and the future. One related species, *Cupressus chengiana*, in the eastern Qinghai–Tibet plateau experienced a population contraction event during the late Quaternary (Xu et al., 2010). Rare and relict plant species such as *Ginkgo biloba*, *Davidia involucrate*, *Liriodendron chinense*, *Camptotheca acuminate*, and *Taxus chinensis* are found in the distribution area of *T. sutchuenensis* (Ma et al., 2017). Cool and dry climate in the Late Pliocene (Li et al., 2004), acting as a vicariance driver, forced a southward migration of *Thuja* in Shanxi province and survived in the Daba mountains (Cui et al., 2015). The relatively stable habitats within the Qinling–Daba Mountains have served as a refugia for more than 1,620 endemic plants in China (Ying, 1994), which have been supported by other plant species such as *Cathaya argyrophylla* (Wang and Ge, 2006), *Saruma henryi* (Zhou et al., 2010), and tree peony species (Xu et al., 2019).

Narrow-ranged species generally exhibited lower genetic diversity than widespread congeners (Cole, 2003). In this study, however, we found the cpSSR genetic diversity of *T. sutchuenensis* was not lower (*He* = 0.23–0.53) than other narrow-ranged species such as the *Camellia huana* (*He* = 0.339–0.605 for cpSSR, *Ho* = 0.353–0.605 for cpSSR) (Li et al., 2020), or widespread species, for example, *Pinus kesiya* (*He* = 0.654 for cpSSR) (Rai and Ginwal, 2018) and *Ambrosia trifida* (*He* = 0.203–0.645 for cpSSR) (Sharma et al., 2020). *T. sutchuenensis* have comparable genetic diversity with the other two congener species, *T. koraiensis* (SSR *He* = 0.548) and *T. occidentalis* (SSR *He* = 0.574–0.624) (Xu et al., 2013; Hou et al., 2018). This relatively high cpSSR genetic diversity of *T. sutchuenensis* might be due to current individuals from large populations that existed before in northern China, which indicates the likely effects of evolutionary history (from Northern China to the Daba Mountains) on genetic diversity. An alternative explanation is that chloroplast genomic inheritance is conservative and maintain genetic material, and chloroplast genome is mainly paternally inherited in Cupressaceae (Sakaguchi et al., 2014).

However, the nucleotide genetic diversity (*pi* = 0.082–0.091 for variant positions, *pi* = 0.0053–0.0062 for all positions) of *T. sutchuenensis* in this study is relatively low compared with other narrow-ranged species, such as *Tetraena mongolica* (*He* = 0.337–0.341; *pi* = 0.349–0.354) based on nRAD-seq (Cheng et al., 2020), or widespread species, such as *Amorphophallus paeoniifolius* (*pi* = 0.072–0.285) (Gao et al., 2017). Nuclear genetic diversity of *T. sutchuenensis* in our study was higher than six single-copy nuclear loci (*pi* = 0.00219) (Qin et al., 2020). Qin et al. (2020) reviewed 12 species of Cupressaceae and the nuclear genetic diversity was 0.00156–0.00989 with 6–13 loci (see Table 4 of Qin et al., 2020). Thus, methods (e.g., SSR, ISSR, and RAD) and the number of loci used to calculate genetic diversity influence the values of genetic diversity. For example, endemic species showed lower genetic diversity (*Ho*, *He*) within populations than regional or widespread species using STMS (sequence-tagged microsatellite sites)-based data, while there is no difference between them using RAPD (random amplified polymorphic DNA)-based data (Nybom, 2004). In addition, the genetic diversity of different species depends on their life form, breeding system, and geographic range (Nybom, 2004).

Generally, low genetic diversity result from high genetic differentiation and low gene flow, but the genetic differentiation was low between *T. sutchuenensis* populations (*F*_*ST*_ < 0.2), with high gene flow. This might result from the low population size of *T. sutchuenensis* (∼10,000 wild individuals in the nature). It is consistent with other narrow-ranged species, for example, the Cupressaceae species *Platycladus orientalis* (*F*_*ST*_ = 0.024–0.386) (Chang et al., 2019), endangered *Camellia huana* (FST = 0.2159 for cpSSR) (Li et al., 2020), threatened *Rhododendron cyanocarpum* (*F*_*ST*_ = 0.0314–0.0452) (Liu et al., 2020), and *Tetraena mongolica* (*F*_*ST*_ = 0.010–0.026) (Cheng et al., 2020). The genetic differentiation between populations was strongly influenced by genetic drift, gene flow, long-term evolution, mating systems, selection, and mutation (Hamrick and Godt, 1996). Due to gymnosperm species having long generation times, they usually show low genetic differentiation from wind pollination-mediated gene flow and breeding systems (Hamrick et al., 1992). *T. sutchuenensis* is a wind-pollinated hermaphrodite plant, and the low genetic differentiation resulted from the long-distance gene dispersal by pollen grains or seeds, because the extant *T. sutchuenensis* populations were geographically close. TreeMix results of nRAD-seq showed migration between close populations (QS and XY), and genetic and geographic distance indicating a weak effect of isolation by distance.

In fact, population structure results based on nRAD-seq of three cultivated and six wild populations failed to reveal any meaningful cluster patterns, which consisted with a previous study (Qin et al., 2020). However, population structure from six wild populations showed three clusters but with extensive gene flow and introgression between clusters. TreeMix results of cpSSR and nRAD-seq showed historic migrations between these wild populations, which is likely with the construction of roads in the valley between mountains (Figure 1D). In Cupressaceae, chloroplast genome is mainly paternally inherited, and compared with microsatellite regions, the low mutation rate of nRAD-seq loci can reveal the ancestral pattern of genetic structure (Andrews et al., 2016). This indicates the importance of constructing corridors in the conservation of genetic diversity (Yu et al., 2020).

In addition, the reintroduction of endangered species is an effective pathway to conserve genetic diversity. It has been demonstrated that reintroduction success can be enhanced by using plant material from large and stable source populations (Godefroid et al., 2011). Structure analysis can find the origination of reintroduced populations, we found that the reintroduced GMR and BQR populations cultivated by a cottage method were from the wild QS population. Interestingly, the reintroduced XYR population developed from the seeds of the XY population 15 years ago did not cluster with other any wild populations. Assessing the genetic diversity and genetic structure of endemic and endangered species is a fundamental criterion in developing scientific and rational conservation strategies. The reintroduction of *T. sutchuenensis* should collect plant material from populations with high genetic diversity such as the QS and BQ populations. In these populations, plant material from different age and size categories should be collected wherever possible in order to sample representative genetic diversity (Lauterbach, 2013).

In conclusion, we found the wild populations of *T. sutchuenensis* in the Daba mountains, i.e., its entire distributed region, were from a common ancestral population at 203 ka, which indicates *T. sutchuenensis* have colonized the Daba mountains before contraction at the Last Interglacial (LIG, ∼120–140 ka). The relatively high chloroplast and low nuclear genetic diversity of *T. sutchuenensis* populations might explain why it can and only survive in the Daba mountains as a “refuge.” After long-term (∼203 ka) adaptation, *T. sutchuenensis* prefers to grow on limestone cliffs, crest ridges, or steep slopes with special climate, soil (pH = 7.53), and sunshine (Ma et al., 2017), which leads to a narrow ecological niche of *T. sutchuenensis* and further to a low nuclear genetic diversity. These restrict its distribution only in the Daba mountains, although the seeds of *T. sutchuenensis* are wind-disseminated with membranous wings that facilitate population spread. Thus, enforcing the reintroduction of plant material from populations with high genetic diversity facilitates the conservation of *T. sutchuenensis*.

## Data Availability

The original contributions presented in the study are publicly available. These data can be found here: PRJNA777040.
