# Supplementary material for: Chloroplast and Nuclear Genetic Diversity Explain the Limited Distribution of Endangered and Endemic Thuja sutchuenensis in China
Source: Front Genet. 2021 Dec 23;12:801229. doi: 10.3389/fgene.2021.801229 (PMC8733598; doi:10.3389/fgene.2021.801229)
Supplement: Supplementary file 1 [file DataSheet1.docx]

**TABLE S1** Primer sequence, product sizes and melting temperature (Tm) of 30 microsatellite markers for analyzing chloroplast genetic diversity

| Locus code | Primer sequences (5’-3’) | Standard length(bp) | Tm | Fluorescent |
| --- | --- | --- | --- | --- |
| TS-F1 | GCTTATGCTGGCTTCCACTT | 100 | 60 | FAM |
|  | CAGAGTTAAGGCCAATGGGA |  |  |  |
| TS-F2 | GACACCCATGGCTCCTTTTA | 127 | 60 | HEX |
|  | GGGGTGCTCTATTCAAATGC |  |  |  |
| TS-F3 | TCGGTTGAGCAGACATTGAG | 148 | 60 | TRAMA |
|  | GTGGCTCCATTTCACAGGTT |  |  |  |
| TS-F4 | TATTATAAGGGCCGGGTTCC | 204 | 60 | ROX |
|  | TGGATGGGAAGGGGTATGTA |  |  |  |
| TS-F5 | ATTGTGAAGGGAGCACAACC | 103 | 60 | FAM |
|  | AAGTGGCAAAGAGAAGGGCT |  |  |  |
| TS-F6 | AACGCTTCACCCAACCTATG | 119 | 60 | HEX |
|  | TTAAGGTGGGTAAAGGCACG |  |  |  |
| TS-F7 | AACCAAGTGCAACCTATGCC | 162 | 60 | TRAMA |
|  | CTCAGCTTACCTTCGTTCGG |  |  |  |
| TS-F8 | CCCTACTCAGAGGCTTGTGC | 248 | 60 | ROX |
|  | CTATGCAGGCATCCAAAGGT |  |  |  |
| TS-F9 | GCTTCTGGTTGAGTTAGCCG | 104 | 60 | FAM |
|  | CAAAGCGACTGGAGACTTCC |  |  |  |
| TS-F10 | GCGTCCAAAAGACAACCTGT | 138 | 60 | HEX |
|  | TGGCAAATTTGTTGGCATTA |  |  |  |
| TS-F11 | GGTCACATGAACACGTCTGG | 168 | 60 | TRAMA |
|  | TAGGGGAGCAGAGTGCAGTT |  |  |  |
| TS-F12 | AGGTACCCATGTTGGTTTCG | 105 | 60 | FAM |
|  | AAGTACCTTGCCACTGGTGC |  |  |  |
| TS-F13 | CGAACAACAGGGAACCAAGT | 129 | 60 | HEX |
|  | AACCCATCCCTTTTATGGCT |  |  |  |
| TS-F14 | CTTTAGGCCAGCAGAACCAG | 170 | 60 | TRAMA |
|  | GGAAGATTGGCTTGGCATTA |  |  |  |
| TS-F15 | CCTCCTCCTCCTCCTCCTTA | 232 | 60 | ROX |
|  | GGGGATGTAGGGACACTCAA |  |  |  |
| TS-F16 | GGAGCTGAGGCAGTTAGTGG | 106 | 60 | FAM |
|  | GAACCTACCGTCGTTGGAAA |  |  |  |
| TS-F17 | GCTCTAGCCCTAACCCTGCT | 143 | 60 | HEX |
|  | CTTCGTCGGTGGAGTAGAGC |  |  |  |
| TS-F18 | CGTTGCCCAGTAGTTTGGAT | 178 | 60 | TRAMA |
|  | CACGCACCATAAATGTCGTC |  |  |  |
| TS-F19 | AGCCAGGGTTGCCCTACTAT | 273 | 60 | ROX |
|  | CGCTTCGAAACTGGAAGAAC |  |  |  |
| TS-F20 | GGGGCTTATCTACTCTGCCC | 106 | 60 | FAM |
|  | CGTCCAGGTAGGTCCAAAAA |  |  |  |
| TS-F21 | ATGTATTTTCTTCCCGTGCG | 144 | 60 | HEX |
|  | TCCCGAAATGTTGGGAATAA |  |  |  |
| TS-F22 | CGGGATGGAGAGATTGAAAA | 181 | 60 | TRAMA |
|  | CGGCAACCAAAGTATGTCCT |  |  |  |
| TS-F23 | GGCAATTTGTCTAACCGCAT | 280 | 60 | ROX |
|  | TGCCCAGATGTTAACGCATA |  |  |  |
| TS-F24 | GTACTTTCACCCGCCCACTA | 110 | 60 | FAM |
|  | GGCATCGATGGGAAGAGATA |  |  |  |
| TS-F25 | GGAAGTGACCACGTCCAGTT | 145 | 60 | HEX |
|  | TATTGGGATCGGAGAAGTCG |  |  |  |
| TS-F26 | TCCACCCCCACTATCCATAA | 185 | 60 | TRAMA |
|  | TATGCCTATGCAGCTTCGTG |  |  |  |
| TS-F27 | TAACAATGTCGCCCACTTGA | 112 | 60 | FAM |
|  | ACAGGGCTGAGAACAGTCGT |  |  |  |
| TS-F28 | CCTATTATTGGCCAGGGGAT | 148 | 60 | HEX |
|  | CGTATCCGGGTGTGCTATCT |  |  |  |
| TS-F29 | GCTCGCACCAGCCTACTATC | 191 | 60 | TRAMA |
|  | AGAAGTTGGAGCCAAAGCAA |  |  |  |
| TS-F30 | ATTTGTGCAGATCTTTGGGG | 273 | 60 | ROX |
|  | AATCCTTTCCATGGTTGCTG |  |  |  |

**TABLE S2** The PCR amplification program for cpSSR

| Steps | Temperature | Time |  |
| --- | --- | --- | --- |
| Pre degeneration | 94℃ | 5 min |  |
| Degeneration | 94℃ | 30 s | 35 Circulation |
| Annealing | 60℃ | 30 s |  |
| Extension | 72℃ | 40 s |  |
| Final extension | 72℃ | 7 min |  |
|  | 4℃ | Pause |  |

**TABLE S3** Testing of linkage disequilibrium among 30 microsatellite loci (significance level at p=0.05)

| locus | 1 | 2 | 3 | 4 | 5 | 6 | 7 | 8 | 9 | 10 | 11 | 12 | 13 | 14 | 15 | 16 | 17 | 18 | 19 | 20 | 21 | 22 | 23 | 24 | 25 | 26 | 27 | 28 | 29 | 30 |
| --- | --- | --- | --- | --- | --- | --- | --- | --- | --- | --- | --- | --- | --- | --- | --- | --- | --- | --- | --- | --- | --- | --- | --- | --- | --- | --- | --- | --- | --- | --- |
| 1 | * | + | + | + | + | + | + | + | + | + | + | + | + | + | + | + | + | + | + | + | + | + | + | + | + | + | + | + | + | + |
| 2 | + | * | + | + | + | + | + | + | + | + | + | + | + | + | + | + | + | + | + | + | + | + | + | + | + | + | + | + | + | + |
| 3 | + | + | * | - | + | + | + | + | + | + | + | + | + | + | + | + | + | + | + | + | + | + | + | + | + | + | + | + | + | + |
| 4 | + | + | - | * | + | + | + | + | + | + | + | + | + | + | + | - | + | + | + | + | + | + | - | + | + | + | + | + | + | + |
| 5 | + | + | + | + | * | + | + | + | + | + | + | + | + | + | + | + | + | + | + | + | + | + | + | + | + | + | + | + | + | + |
| 6 | + | + | + | + | + | * | + | + | + | + | + | + | + | + | + | + | + | + | + | + | + | + | + | + | + | + | + | + | + | + |
| 7 | + | + | + | + | + | + | * | + | + | + | + | + | + | + | + | + | + | + | + | + | + | + | + | + | + | + | + | + | + | + |
| 8 | + | + | + | + | + | + | + | * | + | + | + | + | + | + | + | + | + | + | + | + | + | + | + | + | + | + | + | + | + | + |
| 9 | + | + | + | + | + | + | + | + | * | + | + | + | + | + | + | + | + | + | + | + | + | + | + | + | + | + | + | + | + | + |
| 10 | + | + | + | + | + | + | + | + | + | * | + | + | + | + | + | + | + | + | + | + | + | + | + | + | + | + | + | + | + | + |
| 11 | + | + | + | + | + | + | + | + | + | + | * | + | + | + | + | + | + | + | + | + | + | + | + | + | + | + | + | + | + | + |
| 12 | + | + | + | + | + | + | + | + | + | + | + | * | + | + | + | + | + | + | + | + | + | + | + | + | + | + | + | + | + | + |
| 13 | + | + | + | + | + | + | + | + | + | + | + | + | * | + | + | + | + | + | + | + | + | + | + | + | + | + | + | + | + | + |
| 14 | + | + | + | + | + | + | + | + | + | + | + | + | + | * | + | + | + | + | + | + | + | + | + | + | + | + | + | + | + | + |
| 15 | + | + | + | + | + | + | + | + | + | + | + | + | + | + | * | + | + | + | + | + | + | + | + | + | + | + | + | + | + | + |
| 16 | + | + | + | - | + | + | + | + | + | + | + | + | + | + | + | * | + | + | + | + | + | + | + | + | + | + | + | + | + | + |
| 17 | + | + | + | + | + | + | + | + | + | + | + | + | + | + | + | + | * | + | + | + | + | - | + | + | + | + | + | + | + | + |
| 18 | + | + | + | + | + | + | + | + | + | + | + | + | + | + | + | + | + | * | + | - | + | + | + | + | + | + | + | + | + | + |
| 19 | + | + | + | + | + | + | + | + | + | + | + | + | + | + | + | + | + | + | * | + | - | + | + | - | + | + | + | + | + | + |
| 20 | + | + | + | + | + | + | + | + | + | + | + | + | + | + | + | + | + | - | + | * | + | + | + | + | + | + | + | + | + | + |
| 21 | + | + | + | + | + | + | + | + | + | + | + | + | + | + | + | + | + | + | - | + | * | + | + | + | + | + | + | + | + | + |
| 22 | + | + | + | + | + | + | + | + | + | + | + | + | + | + | + | - | + | + | + | + | + | * | + | + | + | + | + | + | + | + |
| 23 | + | + | + | - | + | + | + | + | + | + | + | + | + | + | + | + | + | + | + | + | + | + | * | + | + | + | + | + | + | + |
| 24 | + | + | + | + | + | + | + | + | + | + | + | + | + | + | + | + | + | + | - | + | + | + | + | * | + | + | + | + | + | + |
| 25 | + | + | + | + | + | + | + | + | + | + | + | + | + | + | + | + | + | + | + | + | + | + | + | + | * | + | + | + | + | + |
| 26 | + | + | + | + | + | + | + | + | + | + | + | + | + | + | + | + | + | + | + | + | + | + | + | + | + | * | + | + | + | + |
| 27 | + | + | + | + | + | + | + | + | + | + | + | + | + | + | + | + | + | + | + | + | + | + | + | + | + | + | * | + | + | + |
| 28 | + | + | + | + | + | + | + | + | + | + | + | + | + | + | + | + | + | + | + | + | + | + | + | + | + | + | + | * | + | + |
| 29 | + | + | + | + | + | + | + | + | + | + | + | + | + | + | + | + | + | + | + | + | + | + | + | + | + | + | + | + | * | + |
| 30 | + | + | + | + | + | + | + | + | + | + | + | + | + | + | + | + | + | + | + | + | + | + | + | + | + | + | + | + | + | * |

The symbol “+” means no significant linkage disequilibrium between loci.

**TABLE S4** The 19 Bioclimatic factors

| Variable | Description | Variable | | Description |
| --- | --- | --- | --- | --- |
| Bio 1 | Mean annual temperature | Bio 11 | | Mean temperature of coldest quarter |
| Bio 2 | Mean diurnal range | Bio 12 | | Annual precipitation |
| Bio 3 | Isothermality | Bio 13 | | Precipitation of wettest month |
| Bio 4 | Temperature seasonality | Bio 14 | | Precipitation of driest month |
| Bio 5 | Max temperature of warmest month | Bio 15 | | Precipitation seasonality |
| Bio 6 | Min temperature of coldest month | Bio 16 | | Precipitation of wettest quarter |
| Bio 7 | Temperature annual range | Bio 17 | | Precipitation of driest month |
| Bio 8 | Mean temperature of wettest quarter | Bio 18 | | Precipitation of warmest quarter |
| Bio 9 | Mean temperature of driest quarter | Bio 19 | | Precipitation of coldest quarter |
| Bio 10 | Mean temperature of warmest quarter | |  |  |

**TABLE S5** Testing of departure from Hardy-Weinberg equilibrium (significance level at p=0.05)

| Population | BQ | MZ | GM | XY | QS | MY | GMR | BQR | XYR |
| --- | --- | --- | --- | --- | --- | --- | --- | --- | --- |
| TS-F1 | - | - | - | - | - | - | - | - | - |
| TS-F2 | - | - | - | + | + | - | - | - | + |
| TS-F3 | + | - | + | - | - | - | - | + | + |
| TS-F4 | - | - | - | - | - | - | - | - | - |
| TS-F5 | - | - | - | - | - | - | - | - | - |
| TS-F6 | - | - | - | - | - | + | - | - | + |
| TS-F7 | - | - | - | - | - | - | - | - | - |
| TS-F8 | - | - | + | + | - | - | - | - | + |
| TS-F9 | - | - | - | - | - | - | - | - | - |
| TS-F10 | - | - | - | + | - | - | - | - | + |
| TS-F11 | - | - | - | - | - | - | - | - | - |
| TS-F12 | - | + | + | + | - | - | - | - | + |
| TS-F13 | - | - | - | - | - | - | - | - | - |
| TS-F14 | - | - | + | - | + | - | - | + | - |
| TS-F15 | + | + | + | + | - | + | - | - | + |
| TS-F16 | - | - | + | + | + | - | + | + | - |
| TS-F17 | - | - | - | - | - | - | - | - | - |
| TS-F18 | - | - | - | - | - | + | - | + | + |
| TS-F19 | - | - | - | - | - | + | - | - | - |
| TS-F20 | + | + | + | + | - | + | + | + | - |
| TS-F21 | - | - | - | - | - | - | - | - | + |
| TS-F22 | - | - | - | - | - | - | - | - | - |
| TS-F23 | + | - | + | - | - | - | + | + | + |
| TS-F24 | - | - | + | + | - | - | - | + | - |
| TS-F25 | + | - | + | + | + | - | - | + | + |
| TS-F26 | - | - | - | + | - | - | - | - | - |
| TS-F27 | - | - | - | + | - | - | - | - | - |
| TS-F28 | - | + | - | + | - | - | - | - | - |
| TS-F29 | - | - | - | - | - | - | - | - | - |
| TS-F30 | + | + | - | - | - | - | - | - | - |
| Percentage of loci deviated from HWE | 20% | 17% | 33% | 40% | 13% | 17% | 10% | 27% | 37% |

The symbol “+” means departure from Hardy-Weinberg equilibrium in the locus.

**TABLE S6** Bottleneck analysis of *T. sutchuenensis* populations

| Population | Two-phase mutation model | | | |
| --- | --- | --- | --- | --- |
|  | Wilcoxon sign rank test | | | Mode-shift test |
| Wild populations | | | | |
| BQ | | 0.2579 | L-shaped | |
| MZ | 0.5169 | | | L-shaped |
| XY | 0.9890 | | | L-shaped |
| GM | 0.9851 | | | L-shaped |
| QS | 0.0015 | | | shifted |
| MY | 0.1645 | | | L-shaped |
| Cultivated populations | | | | |
| GMR | ＜0.001 | | | shifted |
| XYR | 0.9808 | | | L-shaped |
| BQR | 0.0440 | | | shifted |

L-shaped means that population is under mutation-drift equilibrium, indicating no recent bottleneck, while shifted means that population is departure from mutation-drift equilibrium, indicating recent bottleneck.

**TABLE S7** Analysis of molecular variance (AMOVA) for *T. sutchuenensis* populations based on cpSSR and nRAD-seq

| Source of variation | df | sum of squares | variance components | Percentage of variation |
| --- | --- | --- | --- | --- |
| cpSSR | | | | |
| Among populations | 8 | 525.725 | 1.489 | 19% |
| Within populations | 351 | 2158.850 | 6.151 | 81% |
| Total | 359 | 2684.575 | 7.640 |  |
| nRAD-seq | | | | |
| Among populations | 1 | 19.024 | -0.016 | -0.06 |
| Within populations | 268 | 6717.472 | 25.035 | 100.06 |
| Total | 269 | 6736.496 | 25.019 |  |

**TABLE S8** The results of effective population size from Fastsimcoal analysis

| NPOP1 | NPOP2 | NPOP3 |
| --- | --- | --- |
| BQ+XY | MZ+QS | MY+GM |
| 61 | 74919 | 77912 |


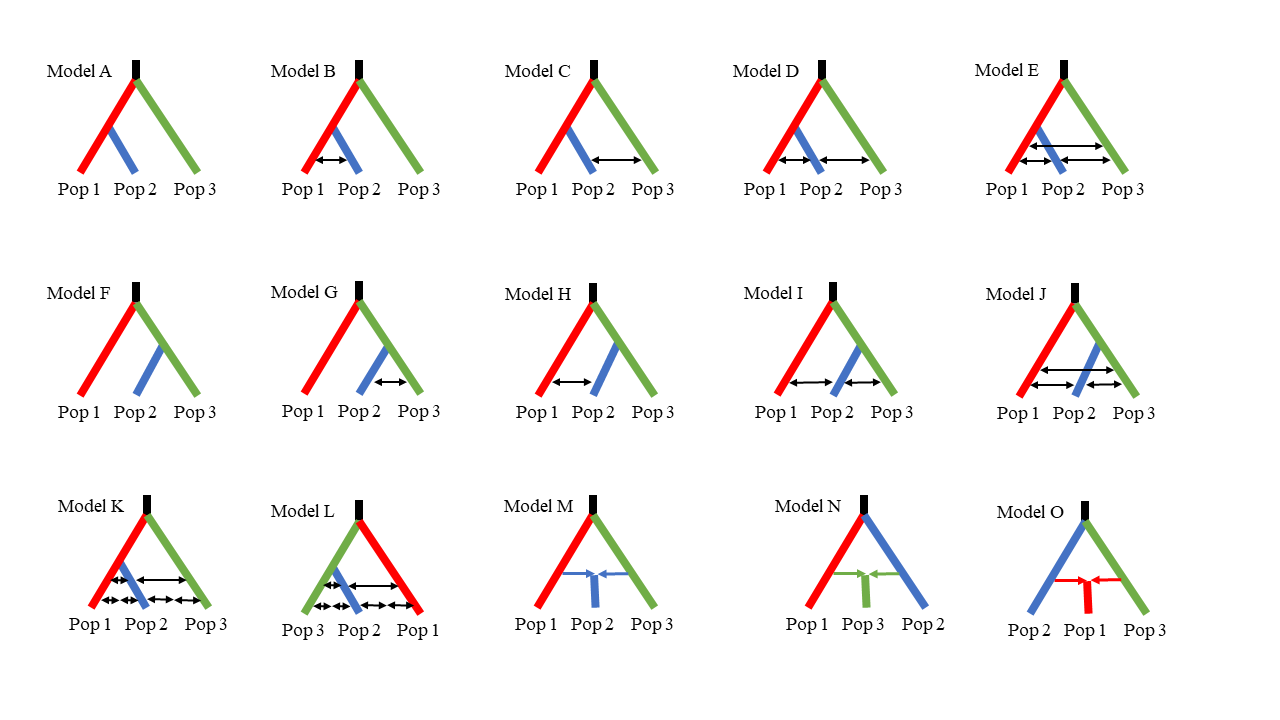


**FIGURE S1** Gene flow models simulated for Fastsimcoal. Abbreviation: Pop 1, BQ and XY; Pop 2, MZ and QS; Pop 3, MY and GM.


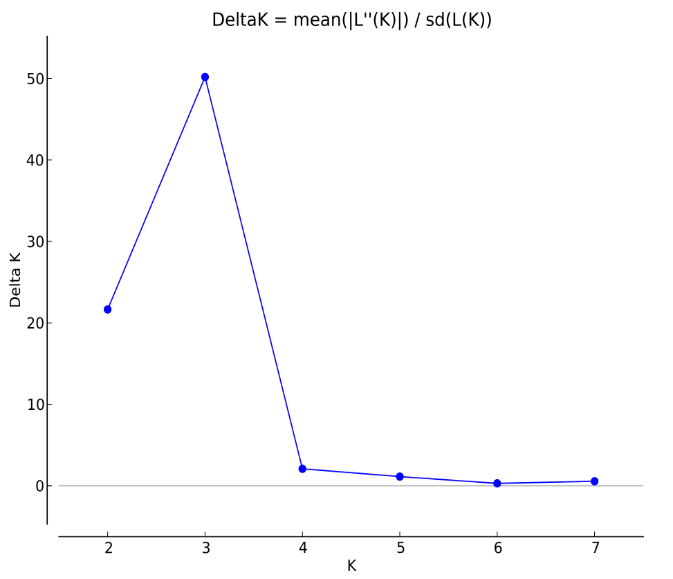


**FIGURE S2** The best K was estimated from the ΔK statistics using Structure Harvester for six wild *T. sutchuenensis* populations. Population structure bar plots from nRAD data showed three clusters (best delta-K=3).


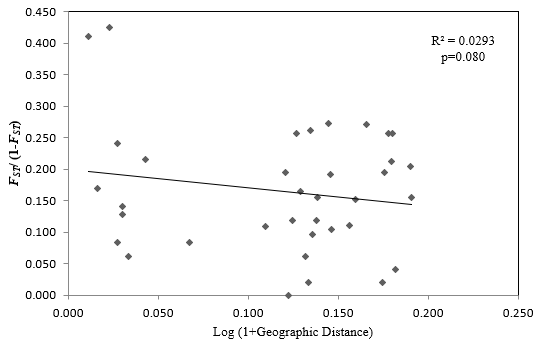


**FIGURE S3** Relationship between genetic differentiation [*F_ST_*/ (1-*F_ST_*)] and geographical distance (log-transformed) between *T. sutchuenensis* populations.


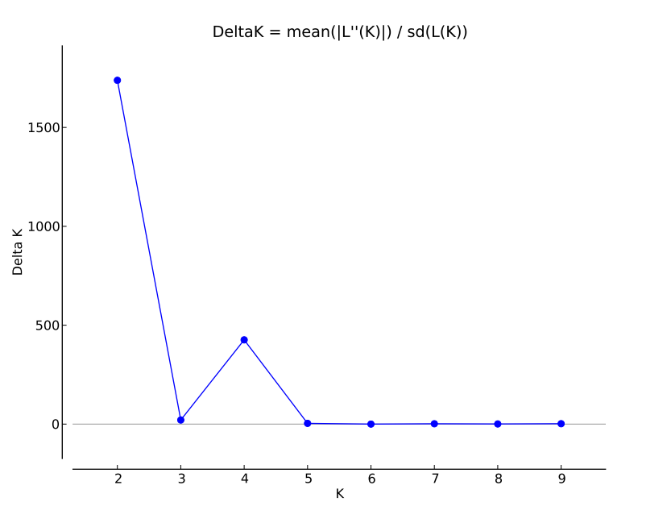


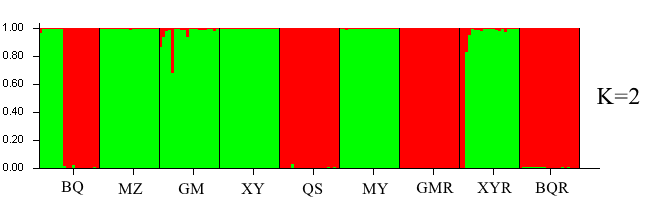


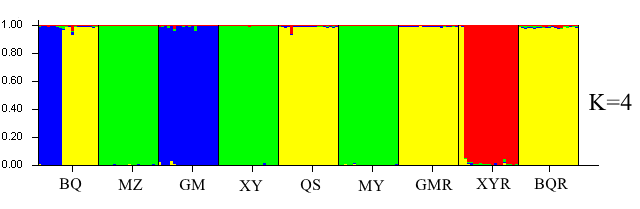


**FIGURE S4** Genetic structure of six wild and three cultivated *T. sutchuenensis* populations. Population structure bar plots from cpSSR show the clustering of samples into 4 clusters (best delta-K=2). Each vertical bar indicates an individual, and the height of each colored bar represents the proportion of assignment to that cluster. Population abbreviations please see Figure 1 and Supplementary Table S1.


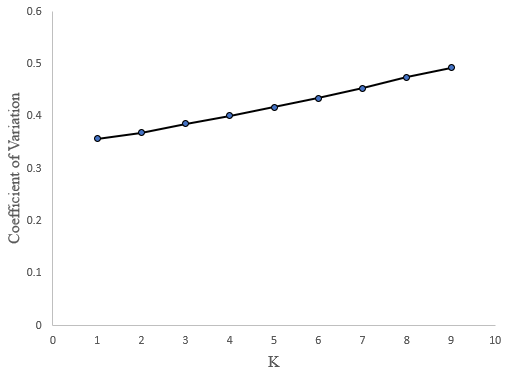


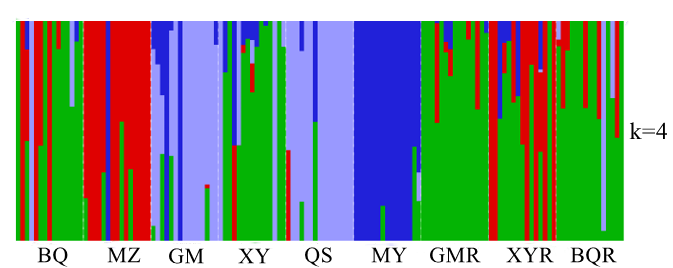


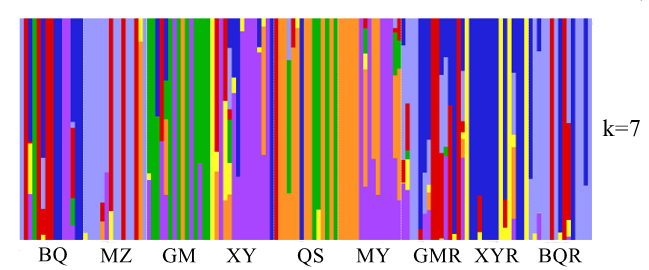


**FIGURE S5** Genetic structure of six wild and three cultivated *T. sutchuenensis* populations. Population structure bar plots from nRAD-seq data show the clustering of samples into 4 clusters (best delta-K=7). Each vertical bar indicates an individual, and the height of each colored bar represents the proportion of assignment to that cluster. Population abbreviations please see Figure 1 and Supplementary Table S1.


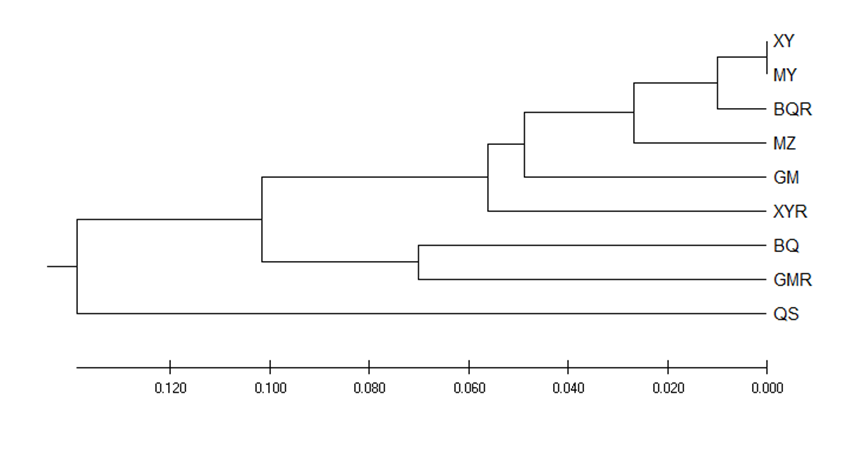


**FIGURE S6** UPGMA dendrograms show the relationships between six wild and three cultivated *T. sutchuenensis* populations with cpSSR. Population abbreviations please see Supplementary Table S1. The third and fourth letters indicate the population abbreviations in the bottom subfigure..


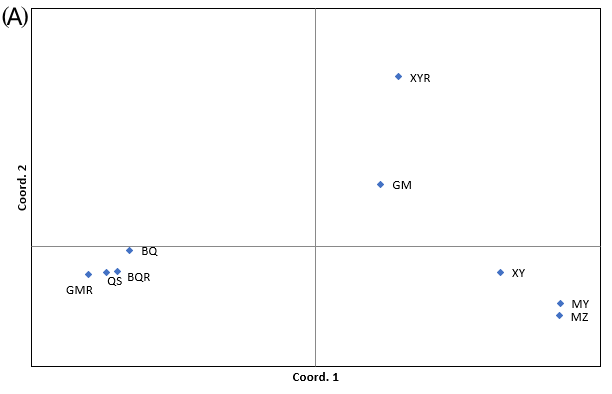


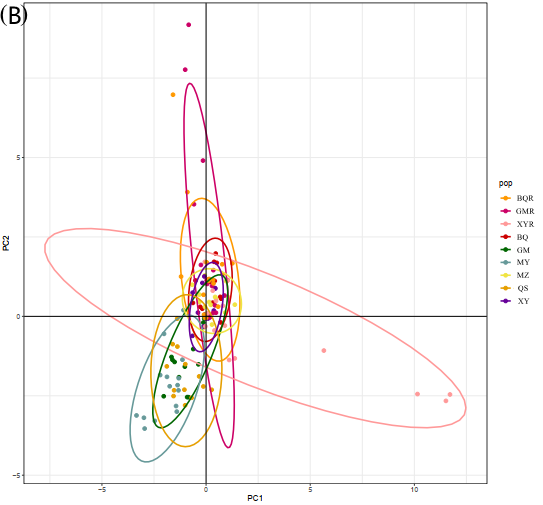


**FIGURE S7** Population principal coordinate analysis (PCoA) for six wild and three cultivated *T. sutchuenensis* populations from cpSSR results (A) and principal component analysis (PCA) from nRAD-seq results (B). Population abbreviations please see Supplementary Table S1.


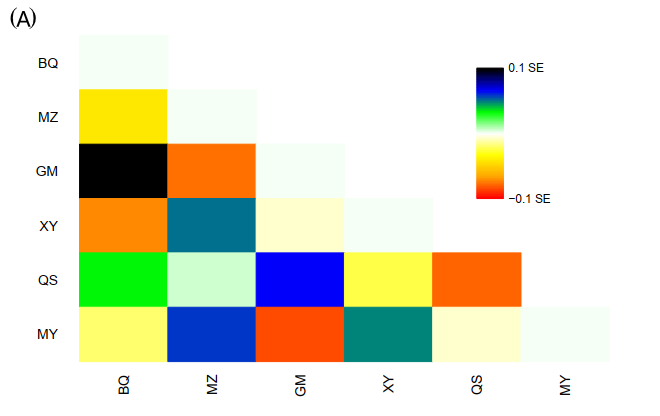

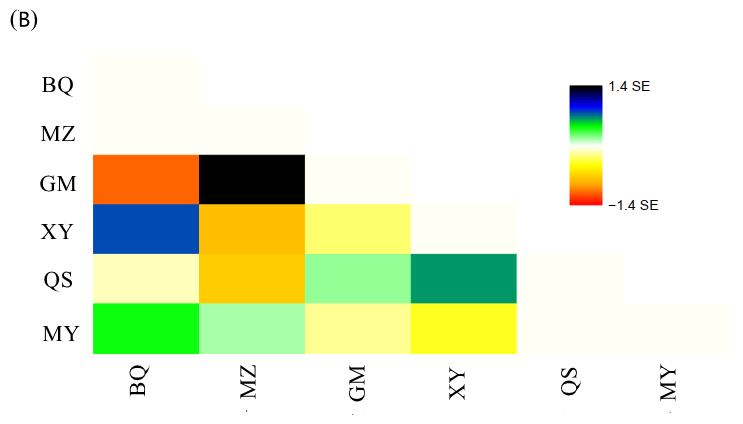


**FIGURE S8** Residual fit plots in the TREEMIX analysis. The residual fit plots based on cpSSR results (A) and nRAD-seq result (B) from the maximum likelihood tree in TREEMIX results. We divided the residual covariance between each pair of populations by the average standard error across all pairs. We then plot in each cell this scaled residual. Colors are described in the palette on the right. Residuals above zero represent populations that are more closely related to each other in the data than in the best-fit tree, and thus are candidates for admixture events.


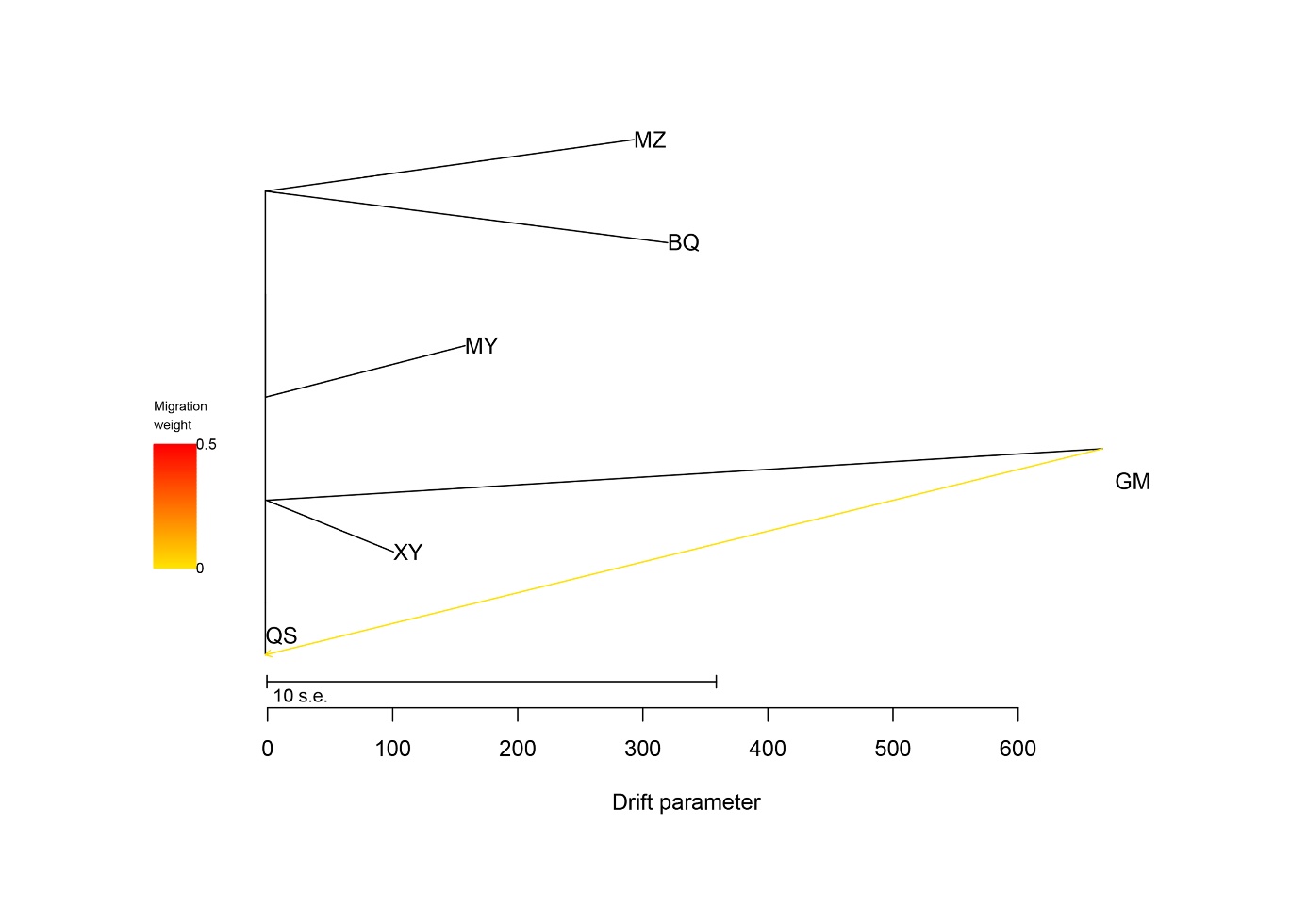

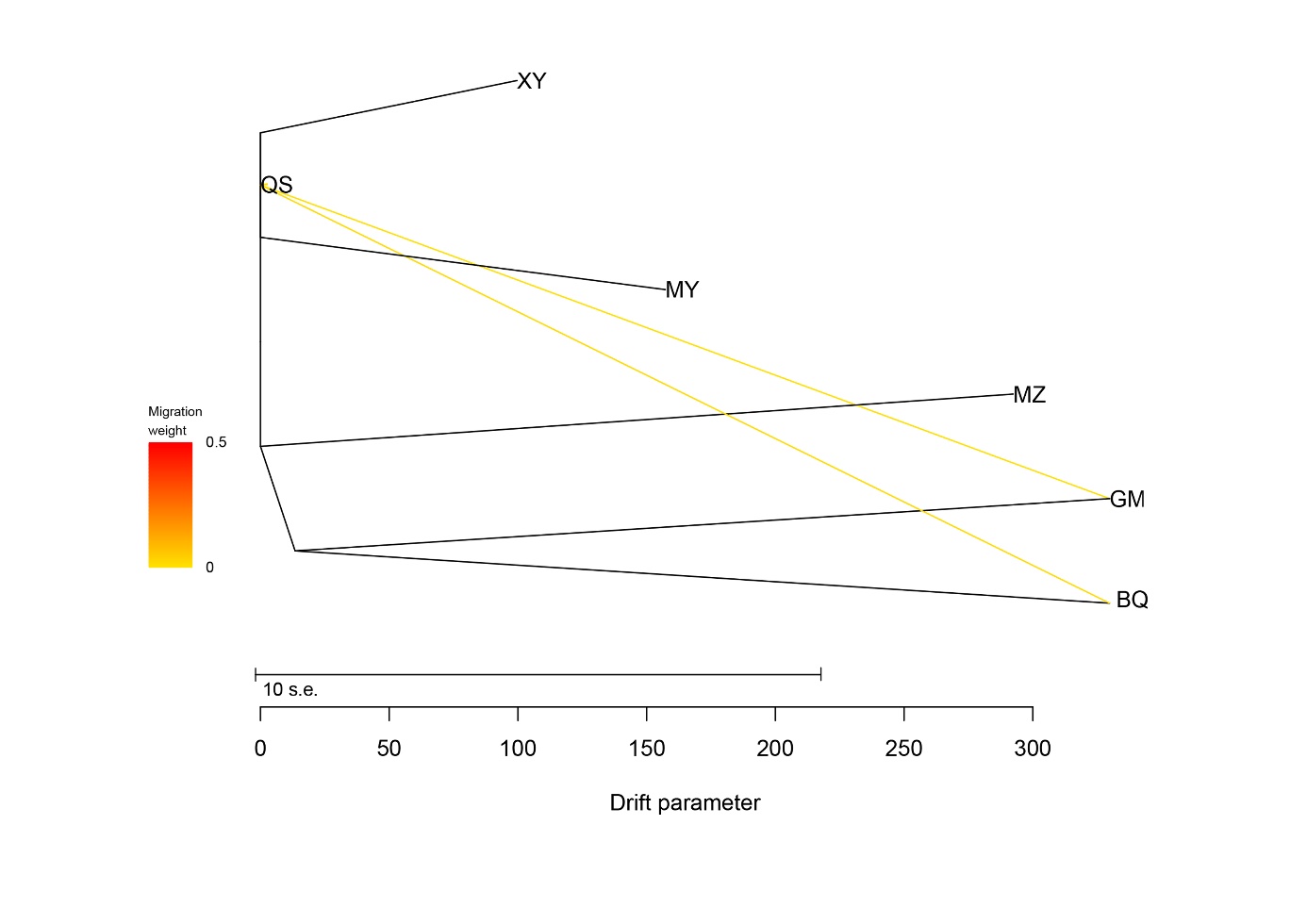


**FIGURE S9** TREEMIX results of cpSSR showing historical mitigation between the wild *T. sutchuenensis* populations (m=1 and m=2). Population abbreviations please see Supplementary Table S1.


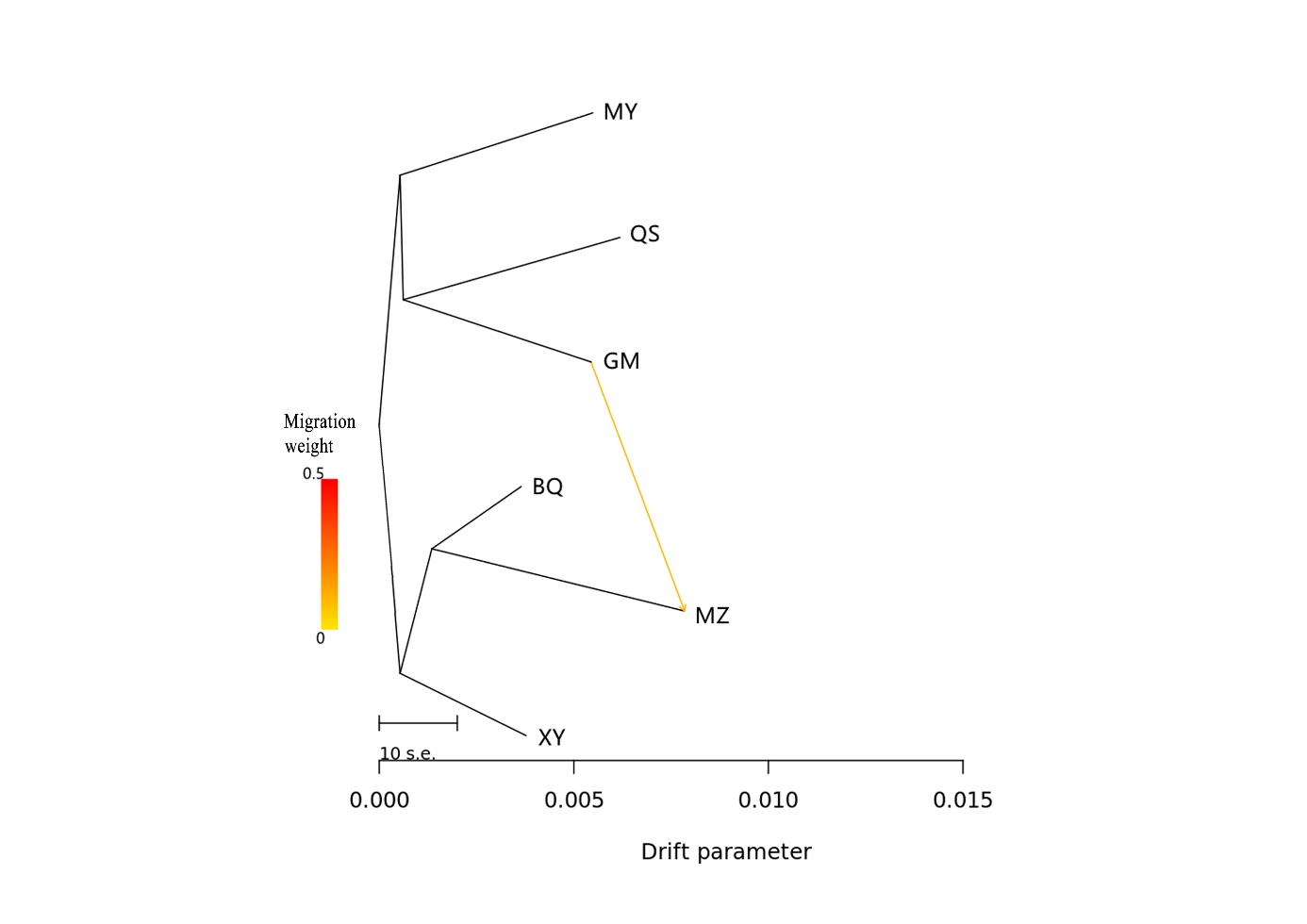

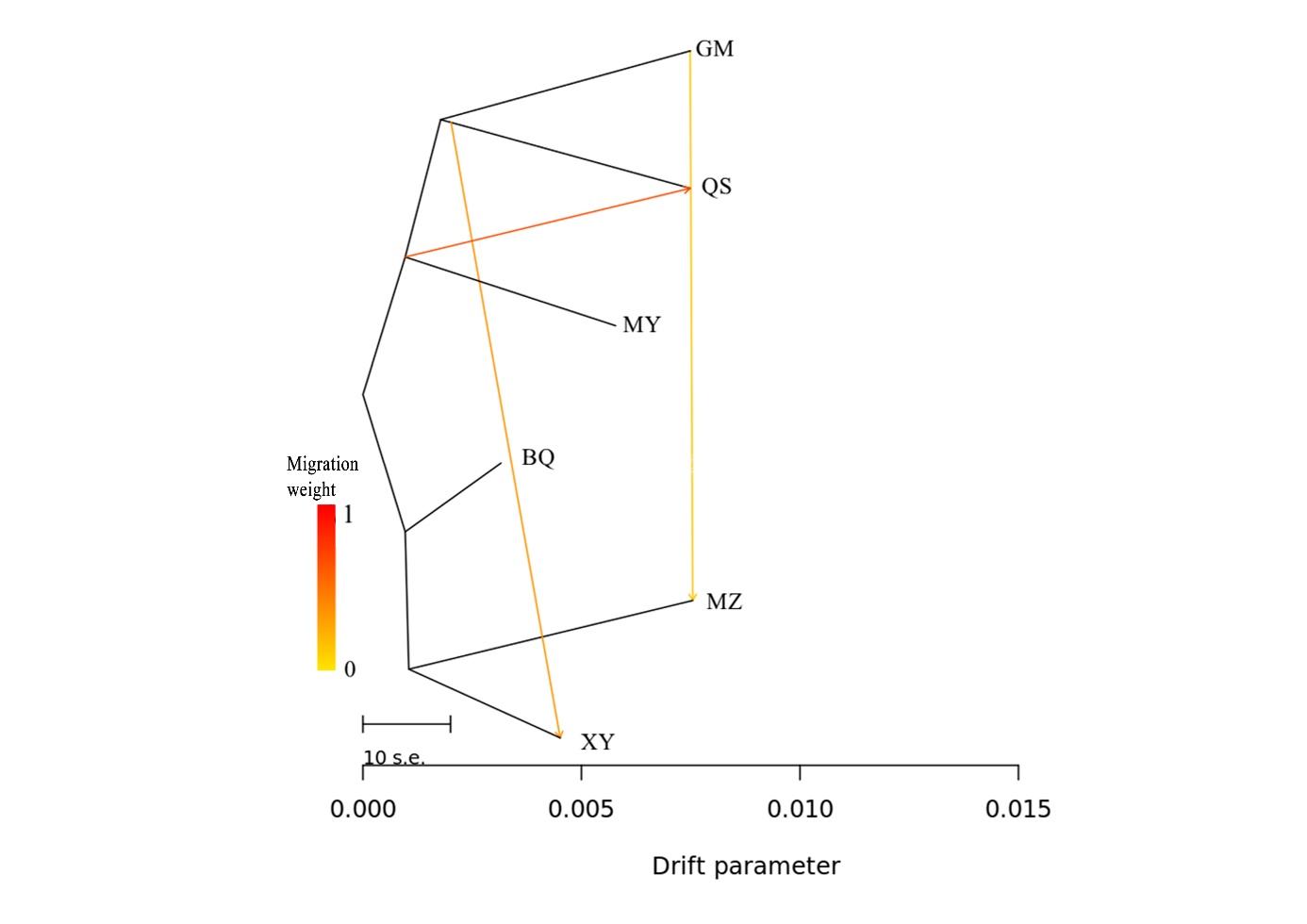
 **FIGURE S10** TREEMIX results of nRAD-seq showing historical mitigation between the wild *T. sutchuenensis* populations (m=1 and m=3).
